# Assessment of prescriber adherence to guideline-directed medical therapy for heart failure at Jimma Medical Center, Ethiopia

**DOI:** 10.1016/j.ijcrp.2025.200555

**Published:** 2025-12-05

**Authors:** Getachew Yitayew Tarekegn, Legese Chelkeba, Mariam Dubale Deko, Fisseha Nigussie Dagnew, Samuel Berihun Dagnew, Tilaye Arega Moges, Sisay Sitotaw Anberbr, Taklo Simeneh Yazie, Addisu Assfaw Ayen, Behailu Terefe Tesfaye

**Affiliations:** a Department of Pharmacy, College of Health Science, Debre Tabor University, Debre Tabor, Ethiopia; b Department of Pharmacology and Clinical Pharmacy, College of Health Sciences, Addis Ababa University, Addis Ababa, Ethiopia; c School of Pharmacy, Department of Clinical Pharmacy, Institute of Health, Jimma University, Jimma, Ethiopia; d Department of Clinical Pharmacy, School of Pharmacy, College of Medicine and Health Science, University of Gondar, Gondar, Ethiopia; e Department of Internal Medicine, School of Medicine, College of Health Sciences, Debre Tabor University, Debre Tabor, Ethiopia

**Keywords:** Heart failure, Reduced ejection fraction, GDMT, Prescription adherence, Patient adherence, Ethiopia

## Abstract

**Background:**

Heart failure with reduced ejection fraction (HFrEF) requires guideline-directed medical therapy (GDMT) to reduce morbidity and mortality. However, adherence to GDMT is often suboptimal in resource-limited settings, such as the Jimma Medical Center, Ethiopia.

**Objectives:**

To assess prescriber adherence to guideline-based medical therapy in heart failure at Jimma Medical Center, Ethiopia.

**Methods:**

A convergent mixed-methods study was conducted from December 2023 to April 2024, enrolling 215 adult HFrEF patients. Prescriber adherence was measured using the Guideline Adherence Index (GAI) and QUALIFY scores. Semi-structured interviews with physicians explored the qualitative factors that affect adherence. Multinomial logistic regression identified the factors associated with adherence.

**Results:**

Only 13.5 % of patients were discharged on all four recommended GDMT classes (ACEI/ARB, beta-blockers, MRAs, and SGLT2 inhibitors). Moderate and poor prescriber adherence occurred in 70.1 % and 16.3 % of the cases, respectively. Patient adherence was higher (74.9 % by MARS-5), yet 94 % were physically inactive, and 54.4 % regularly consumed salt. Insurance coverage (AOR: 4.81; p = 0.001), salt restriction (AOR: 6.62; p = 0.003), and shorter hospital stays (AOR: 0.90; p = 0.005) were linked to better adherence, whereas higher ejection fraction (AOR: 1.07; p = 0.005) and more comorbidities (AOR: 2.36; p = 0.037) predicted poorer adherence. The qualitative findings highlighted medication stock-outs, financial barriers, and clinical complexities as key challenges, with institutional support and multidisciplinary care as facilitators.

**Conclusions:**

Prescriber and patient adherence to GDMT for HFrEF at Jimma Medical Center was suboptimal. Interventions targeting prescriber training, expansion of insurance coverage, patient education, and multidisciplinary care are needed to improve outcomes.

## 1 Introduction

Heart failure with reduced ejection fraction (HFrEF) constitutes a major and escalating global health challenge, affecting an estimated 64 million individuals worldwide [1]. Characterized by impaired left ventricular systolic function, HFrEF leads to significant morbidity, mortality, and frequent hospitalizations, placing a substantial burden on healthcare systems, particularly in low- and middle-income countries (LMICs) [2]. In Ethiopia, the prevalence of cardiovascular diseases is rising, with heart failure emerging as a leading cause of hospital admissions and death [3,4]. Therefore, the effective management of HFrEF is critical to mitigating its adverse health and economic impacts on resource-constrained settings.

Guideline-directed medical therapy (GDMT) is the cornerstone of evidence-based management for HFrEF. Contemporary clinical guidelines, including the 2023 European Society of Cardiology (ESC) recommendations, advocate the use of four key pharmacological classes: angiotensin-converting enzyme inhibitors (ACEIs) or angiotensin receptor blockers (ARBs), beta-adrenergic blockers, mineralocorticoid receptor antagonists (MRAs), and sodium-glucose cotransporter-2 inhibitors (SGLT2i) [5]. These therapies have consistently demonstrated reductions in mortality and hospital readmissions, alongside improved quality of life [6,7]. Despite these clear benefits, adherence to GDMT remains suboptimal globally, with particularly pronounced gaps in LMICs due to challenges such as limited drug availability, financial barriers, and constrained healthcare infrastructure [8,9].

While the efficacy of GDMT is unequivocal, limited data exist regarding prescriber adherence to these guidelines in resource-limited settings such as Ethiopia [10]. Furthermore, the multifactorial influences on prescribing behavior, including medication stock-outs, provider knowledge, and institutional support, remain poorly characterized [11]. Patient adherence to pharmacotherapy and lifestyle modifications is similarly underexplored. These knowledge gaps restrict effective interventions to improve heart failure care and outcomes.

Suboptimal prescriber and patient adherence to GDMT significantly contributes to adverse clinical outcomes, including increased mortality and recurrent hospitalizations. In Ethiopia, the magnitude and determinants of these adherence gaps have not been comprehensively assessed. Addressing this deficit is critical to inform strategies that optimize guideline implementation and improve patient outcomes in similar contexts.

This study aimed to quantitatively evaluate prescriber adherence to GDMT among hospitalized HFrEF patients, assess patient adherence to prescribed medications and lifestyle recommendations, and identify associated clinical, patient-related, and healthcare system factors. Additionally, through qualitative inquiry, it seeks to explore healthcare providers’ perspectives on barriers and facilitators influencing guideline adherence.

By providing a holistic understanding of adherence patterns and challenges, this mixed-methods study will generate evidence to guide policy development, clinical practice improvements, and targeted interventions. Ultimately, these efforts aim to enhance the quality of heart failure management and reduce the burden of HFrEF in Ethiopia and comparable resource-limited settings.

## 2 Methods

### 2.1 Study setting, design, and period

A convergent mixed-methods study at Jimma Medical Center (JMC), an 800-bed tertiary referral hospital in Southwest Ethiopia, was conducted from December 1, 2023, to April 30, 2024. JMC serves a catchment area of approximately 15 million people and is the sole referral center in the region. The research integrated a quantitative cross-sectional assessment of physician adherence to guideline-directed medical therapy (GDMT) for patients with heart failure with a qualitative exploration of physicians’ views on the barriers and facilitators to adherence.

### 2.2 Quantitative component

#### 2.2.1 Study population

The study examined 215 adult patients with heart failure and reduced ejection fraction (HFrEF) at JMC, defined by a left ventricular ejection fraction (LVEF) of 50 %. Eligible participants had stable heart failure therapy for a minimum of 4 weeks prior to data collection, while patients with acute reversible heart failure causes, preserved ejection fraction, critical illness, cognitive impairment, or those who declined consent were excluded.

#### 2.2.2 Sample size and sampling technique

The sample size for this study was calculated using the single population proportion formula to estimate the proportion of prescription adherence to guideline-directed medical therapy (GDMT) among patients with heart failure. Based on a previously published study reporting a moderate adherence rate of 47 % (p = 0.47) [12], and to achieve a 95 % confidence level (Z = 1.96) with a margin of error of 7 % (d = 0.07), the initial sample size was calculated as follows:

p = Assumed proportion of moderate adherence to GDMT of heart failure = 0.47
1-p = q = 0.53
d = Expected margin of error = 0.07
Z α/2 = 95 %confidence interval (C.I) = 1.96

n=(zα2)2p(1−p)d2

Thus, n0 = ((1.96)2 x (0.47x 0.53)/(0.07)2 = 195.

To account for potential nonresponse or incomplete data, a 10 % contingency was added, increasing the sample size to approximately 215 participants. This final sample size was used in the study to ensure adequate statistical power to estimate the prescription adherence to GDMT among hospitalized heart failure patients at Jimma Medical Center.

#### 2.2.3 Data collection instruments and procedures

Data collection tools were developed in English after reviewing the literature [[13], [14], [15], [16], [17], [18], [19]] and chart reviews. Trained clinical pharmacists extracted data from medical records and prescription charts using standardized forms, focusing on sociodemographic characteristics, clinical parameters, and medication prescriptions at discharge, particularly GDMT classes. Additionally, they assessed dose titration within 4 weeks post-discharge, insurance status, and length of hospital stay. Physician adherence was evaluated using the Guideline Adherence Index (GAI) and QUALIFY scoring systems [20,21]. Drug titration was calculated as the ratio of actual prescribed doses to theoretical guideline doses. All 215 study participants were clinically diagnosed with HF with reduced ejection fraction (HFrEF) based on prior echocardiographic findings or physician documentation. During the current hospitalization, echocardiography was performed for 143 patients to reassess left ventricular function.

### 2.3 Study variables

**Dependent Variable**: The primary dependent variable was the prescriber's prescription pattern among patients with chronic heart failure.

**Independent Variables:** Independent variables were classified into four main categories:

Patient-related factors: Demographic characteristics such as sex, age, residence, education level, employment status, and socioeconomic status. Health status indicators, including comorbidities, risk factors, duration of heart failure, previous hospitalizations, and substance use, were also included.

**Clinical-related factors:** Clinical indicators such as the type of discharge, relevant laboratory values, and clinical signs and symptoms.

Treatment and management-related factors: Variables related to treatment, including the admitting unit, class of heart failure medication regimen, prescription of guideline-directed medical therapy (GDMT) during hospitalization and at discharge, and the initiation and titration of GDMT.

**Healthcare-related factors:** Aspects related to the healthcare system, including payer information, discharge location, outpatient follow-up contacts, follow-up patterns, and adherence to post-discharge treatment.

### 2.4 Methods: assessment of physician adherence to guideline-directed medical therapy

Physician adherence to guideline-directed medical therapy (GDMT) for heart failure with reduced ejection fraction (HFrEF) was evaluated using two complementary approaches:

1. Guideline Adherence Index:
  The GAI quantifies the proportion of eligible patients prescribed all recommended medications without contraindications, based on the 2023 European Society of Cardiology (ESC) heart failure guidelines. The contraindications included symptomatic hypotension, renal dysfunction, hyperkalemia, and drug intolerance. Adherence was categorized as.
  ⁃ Good adherence: GAI = 1 (all eligible medications prescribed)
  ⁃ Moderate adherence: 0.5 < GAI <1
  ⁃ Poor adherence: GAI ≤0.5
2. GDMT Adherence Scoring (Matsukawa et al., 2023):

To provide a more detailed assessment of the prescribing patterns, a structured scoring system adapted from Matsukawa et al. was applied. This system evaluates the prescription of key HFrEF therapies, including ACE inhibitors/ARBs/ARNI, beta-blockers, mineralocorticoid receptor antagonists (MRA), and SGLT2 inhibitors. Each patient received a composite adherence score, reflecting the proportion of guideline-recommended medications appropriately prescribed.

**Rationale:** Using both the GAI and the Matsukawa scoring system allowed for a comprehensive and quantitative evaluation of physician adherence, capturing both overall compliance and class-specific prescription patterns. This approach facilitated the identification of gaps in guideline implementation and informed potential interventions to improve patient care.

### 2.5 Patient adherence measurement

Patient adherence to medications was assessed using the Medication Adherence Report Scale–5 items (MARS-5), a validated self-report questionnaire scoring five items on a 5-point Likert scale (total score 5–25) [22]. Scores ≥20 were considered adherent, while scores below 20 indicated nonadherence. Lifestyle adherence, including salt intake restriction and physical activity, was assessed through structured interviews. Patients reporting adherence to the salt restriction guidelines and engaging in at least 150 min of moderate-intensity physical activity weekly were considered adherent.

### 2.6 Data quality assurance

The data collection instrument was created in English and its content was verified for completeness and consistency. A pre-test was executed with 5 % of the sample size at a health facility to evaluate the instrument's clarity and relevance before the main study. Regular reviews were conducted by the principal investigator for completeness, with the summarization of data occurring on the same day. The questionnaire was meticulously designed to gather all necessary information and was subsequently translated into Afan Oromo and Amharic, followed by back-translation into English.

### 2.7 Data management and statistical analysis

Quantitative data were analyzed using Epidata v4.6 and STATA v17. Descriptive statistics outlined the baseline characteristics and adherence rates. Multinomial logistic regression identified factors linked to physician adherence levels, incorporating variables with p < 0.25 from the bivariate analyses into the multivariate models. Multicollinearity was assessed with variance inflation factors (VIF <3) and the model fit was evaluated.

### 2.8 Qualitative component

#### 2.8.1 Participant selection and data collection

A purposive sample of 12 physicians, including cardiologists, internists, and clinical pharmacists, involved in the management of heart failure at Jimma Medical Center was selected for semi-structured interviews. Participants, each with at least one year of experience, were interviewed in private settings using a guide based on the literature and study objectives. The topics discussed included knowledge of GDMT, prescribing behaviors, barriers to adherence, challenges in dose titration, and practice improvement recommendations. Interviews lasted 30–45 min, were conducted in English or local languages, audio-recorded with consent, transcribed verbatim, and translated into English as needed.

#### 2.8.2 Data management and transcription

The audio recordings were verbatim transcribed and verified for accuracy. Local language transcripts were translated into English while preserving the meaning and context. All transcripts were anonymized for participant confidentiality, and the research team ensured a clear audit trail during data handling.

#### 2.8.3 Data analysis

Thematic analysis was performed using Braun and Clarke's framework. The process involved familiarization with the data, generating the initial codes independently, collating these codes into potential themes, reviewing and refining the themes for consistency, defining and naming the themes, and creating a narrative report with illustrative quotations. Disagreements in coding and theme identification were addressed through discussion and consensus.

#### 2.8.4 Trustworthiness and rigor

To enhance credibility, several strategies were utilized: investigator triangulation with two independent coders, peer debriefing during team discussions, member checking with participants for validation, and maintaining reflexivity to minimize bias. The study also ensured dependability through the documentation of analytic decisions and transferability via detailed descriptions of themes and illustrative quotes.

#### 2.8.5 Operational definitions

• Co-morbidity: The presence of additional medical conditions beyond the primary diagnosis [23].
• GDMT: Heart failure treatments recommended by the ESC guidelines (31).
• Physician adherence: The extent of prescribing recommended GDMT medications, measured by GAI [24].
• Patient adherence: Compliance with prescribed medication regimens and lifestyle recommendations measured by MARS-5 and structured interviews.
• Prescription adherence: Comparison of prescribed medications against guideline recommendations.
• GAI: Percentage of eligible patients prescribed guideline-recommended medications [21].
• Eligibility: Absence of contraindications to medications.
• Tolerance: Medications are considered to be tolerated if patients have acceptable blood pressure, renal function, potassium levels, and absence of adverse effects [24,25].
• Adherence levels: Good (score = 1), moderate (>0.5 to <1), poor (≤0.5) [20].
• Suboptimal therapy: Treatments failing to meet GDMT standards or doses, often due to contraindications, intolerance, or poor adherence, lead to worse outcomes [26,27].
• In this study, heart failure with reduced ejection fraction (HFrEF) was defined as an ejection fraction <50 %, consistent with local clinical practice and previous Ethiopian studies [28,29]. Current international guidelines classify HFrEF as EF <40 % [30,31].

#### 2.8.6 Handling of the missing data

Records with missing or incomplete key data were excluded from the analysis. These accounted for less than 5 % of the sample, minimizing the bias.

#### 2.8.7 Ethical considerations

The study was approved by the Institutional Review Board of Jimma University (Ref. No. JUIH/IRB/694/23). Written informed consent was obtained from the physicians participating in the qualitative interviews. Patient data were collected retrospectively, and confidentiality was maintained. Participation was voluntary, and anonymity was ensured. The study adhered to the Declaration of Helsinki.

## 3 Results

### 3.1 Socio-demographic and behavioral characteristics

There were higher numbers of men (120, 56 %) reported, and the mean age of study participants was 57 years. Patients’ medical insurance coverage was the most common payer at 150 (70 %), followed by out-of-pocket payments at 52 (24 %). Of the levels of education, illiteracy and primary education were nearly equal (illiteracy, 82 (38.1 %)). Most patients reported being physically inactive at 201 (94 %), and the majority of patients consumed salt at 117 (54.4 %). Among the behavioral characteristics, khat chewers were 28 (13 %), and alcohol drinkers were 15 (7 %) (Table 1**).**

**Table 1:** Socio-demographic and behavioral characteristics of heart failure patients admitted to JMC, southwest Ethiopia, from December to April 2024 (N = 215).

Variable	Category	Frequency n (%)
Sex	Male	**120 (55.8)**
	Female	95 (44.2)
Age (years) mean (SD)		**57 (12.3)**
Residence	Rural area	160 (74.4)
	Urban area	55 (25.6)
Admission unit	Cardiac	156 (72.6)
	Medicine ward	53 (24.7)
	Others unit∗	6 (2.8)
Expected primary payer	Insurance	**150 (70)**
	Out of pocket	52 (24)
	Others ∗	13 (6)
employment status	Yes	33 (15.3)
	No	182 (84.7)
Specific occupation	Farmer	87 (40.5)
	Merchant	58 (27)
	Civil servant	17 (7.9)
	Retired	16 (7.4)
	Labor	14 (6.5)
	Student	14 (6.5)
	Self-employed	12 (5.6)
Marital status	Married	142 (66)
	Divorced	33 (15.3)
	Single	27 (12.6)
	Widowed	13 (6)
Education status	Primary education	83 (38.6)
	No formal education	82 (38.1)
	Higher education	38 (17.7)
	Secondary education	12 (5.6)
Family history of heart failure	Yes	12 (5.6)
	No	203 (94.6)
Physical activity∗∗	Yes	14 (6.5)
	No	201 (93.5)
Salt intake	Yes	117 (54.4)
	No	98 (45.6)
Cigarette Smoking	Never	180 (83.7)
	Ex-smoker	34 (15.8)
	Current	1 (0.5)
Khat Chewing	Never	122 (56.7)
	Ex-chewer	60 (30.2)
	Current	28 (13)
Alcohol drinker	Never	130 (60.5)
	Ex-drunk	70 (32.6)
	Current	15 (7)
Total		215 (100)

Key: ∗ Others-from social worker NGO insurance, ∗∗ Physical activity –regular physical activity, s∗s∗s∗ (ex-smoker, ex-drinker, ex-chewer)-not take the last 30 days.

### 3.2 Clinical characteristics and the Framingham criteria

Among the 215 patients with HFrEF, 143 (66.5 %) underwent echocardiography during the current admission, while the remaining had previously documented results confirming reduced ejection fraction. In this cohort, 151 (70.2 %) patients presented with chronic acute decompensated heart failure (ADHF), and 64 (29.8 %) had de novo HF. Most patients had advanced symptoms**,** with NYHA class III in 122 (56.7 %) and class IV in 69 (32.1 %)**,** and only 24 (11.2 %) in class II. Correspondingly, orthopnea (95.8 %)**,** pulmonary rales (85.1 %)**,** and acute pulmonary edema (48.4 %) were highly prevalent, indicating significant congestion. The majority were in ACC/AHA stage C (207, 96.3 %)**.** The median length of hospital stay was 9 days (IQR 6–15) (Table 2).

**Table 2:** Clinical characteristics and Framingham criteria of acute heart failure patients admitted to JMC, southwest Ethiopia, from December to April 2024 (N = 215).

Variable	Category/Finding	Frequency (n)	Percentage (%)
HF Syndrome	De novo HF	64	29.8
	Chronic ADHF	151	70.2
Ejection Fraction (EF)	<30 %	52	24.2
	30–39 %	62	28.8
	40–49 %	29	13.5
NYHA Functional Class	II	24	11.2
	III	122	56.7
	IV	69	32.1
Stage (ACC/AHA)	B	4	1.9
	C	207	96.3
	D	4	1.9
Framingham Major Criteria	Acute pulmonary edema	104	48.4
	Cardiomegaly	133	61.9
	Hepatojugular reflux	11	5.1
	Neck vein distension	39	18.1
	Orthopnea	206	95.8
	Third heart sound	54	25.1
	Weight loss ≥4.5 kg in 5 days	41	19.1
	Pulmonary rales	183	85.1
Framingham Minor Criteria	Ankle edema	211	98.1
	Dyspnea on exertion	193	89.8
	Hepatomegaly	122	56.7
	Nocturnal cough	184	85.6
	Pleural effusion	124	57.7
	Tachycardia (HR ≥ 120 bpm)	13	6.0
Length of hospital stay (median, IQR)	–	9 (6–15)	–

Key: HR, heart rate; IQR, interquartile range; NYHA, New York Heart Association; HF, heart failure; ADHF, acute decompensated heart failure.

### 3.3 Baseline imaging and diagnostic findings of HFrEF

A study of 215 patients with heart failure (HFrEF) found that most had chronic ADHF and advanced symptoms, with orthopnea, pulmonary rales, and acute pulmonary edema being prevalent. The majority were classified as ACC/AHA stage C, with a median hospital stay of 9 days (Table 3).

**Table 3:** Echocardiographic and imaging findings among patients with HFrEF at admission JMC, southwest Ethiopia, from December to April 2024 (N = 215).

Test/Imaging	Finding/Category	Frequency (n)	Percentage (%)
Chest X-ray (N = 193)	Normal	79	40.9
	Pleural effusion	72	37.3
	Pulmonary edema	40	20.7
	Pneumonia	30	15.5
	Cor-pulmonale	14	7.3
	Pleural effusion + pulmonary edema	11	5.7
	Pneumonia + pleural effusion	8	4.1
	Others	2	1.0
Electrocardiography (N = 202)	Normal	109	54.0
	Sinus tachycardia	32	15.8
	Atrial fibrillation	26	12.9
	Heart block (LBBB)	22	10.9
	Acute coronary syndrome	10	5.0
	Ventricular fibrillation	7	3.5
	Ventricular tachycardia	7	3.5
	Bradycardia	3	1.5
	Others	4	2.0
Echocardiography (N = 210)	LVEF <40 %	110	52.4
	Left ventricular hypertrophy (LVH)	37	17.6
	Left ventricular dilation	42	20.0
	Right ventricular dilation	25	11.9
	Right ventricular dysfunction	28	13.3
	Left atrial enlargement (LAE)	33	15.7
	Pulmonary hypertension	29	13.8
	Pericardial effusion	12	5.7
	Others	6	2.9

Footnotes:

⁃ LVEF = Left ventricular ejection fraction; LVH = Left ventricular hypertrophy; LAE = Left atrial enlargement.

⁃ Percentages were calculated as (Frequency/N) × 100.

⁃ Echocardiography findings include only objective structural and functional parameters**,** excluding etiological diagnoses.

### 3.4 Distribution of heart failure etiologies in the study population

Among the 215 patients in the study, ischemic heart disease (IHD) was the leading cause of heart failure, affecting 95 patients (44.2 %). This was followed by valvular heart disease (VHD) in 79 patients (36.7 %), non-ischemic cardiomyopathies in 53 patients (24.7 %), and hypertensive heart disease in 20 cases (9.3 %) (Table 4).

**Table 4:** Etiologies of heart failure (N = 215).

Etiology	Frequency (n)	Percentage (%)
Ischemic heart disease (IHD)	95	44.2
Valvular heart disease (VHD)	79	36.7
Non-ischemic cardiomyopathies	53	24.7
Hypertensive heart disease	20	9.3

Footnotes:

• *IHD: Ischemic heart disease*.

• *VHD: valvular heart disease*.

• *Percentages may sum to more than 100 % if patients have multiple etiologies recorded.*

### 3.5 GDMT prescribing patterns in HF

The prescribing patterns for heart failure medications at the Jimma Medical Center showed suboptimal adherence to guideline-directed medical therapy. Only 13.5 % of patients received all recommended therapies at target doses, with 70.1 % showing moderate adherence and 16.3 % showing poor adherence. The most common combination therapy was ACEI + MRA + beta-blockers, prescribed to 28.4 % of patients. Less frequent combinations included ACEI/ARB/ARNI + SGLT2i (3.3 %), MRA + beta-blockers (3.7 %), ACEI/ARB/ARNI + MRA + SGLT2i (1.9 %), and beta-blockers + SGLT2i (0.5 %) (Figs. 1 and 2*). (Attached in the supplementary material)*.

∗SGLT2i adherence percentage was calculated using only patients eligible to undergo SGLT2i therapy (n = 31) per guideline criteria.

Key: GAI, guideline adherence index; ACEI, converting enzyme inhibitor; ARB, angiotensin receptor blocker; BB, beta blocker; MRA, mineralocorticoid receptor antagonist; ARNI, angiotensin receptor-neprilysin inhibitor; SGLTI, sodium glucose cotransporter-2 inhibitor.

Patients at JMC, Southwest Ethiopia (Dec-Apr 2024, N = 215).

### 3.6 Medication classes prescribed for patients with HFrEF at admission and discharge

In this study on adherence to target doses (TD) for guideline-directed medical therapy (GDMT), varying adherence rates were observed across drug classes. 63.7 % of patients achieved TD for ACE inhibitors, while 36.3 % received less than 50 % due to factors such as up-titration or renal disease. Mineralocorticoid receptor antagonists had a high adherence rate of 97.1 %, but beta-blockers showed lower adherence, with none achieving full TD and 86.3 % receiving under 50 %. All patients on SGLT2 inhibitors reached TD. However, ARNI and SGLT2 inhibitors were underutilized, with only 14.4 % of patients receiving SGLT2 inhibitors. The study attributed suboptimal adherence to issues such as cost, limited availability, and clinical contraindications, aligning with trends in other GDMT drug classes (Table 5).

**Table 5:** Medication classes prescribed for patients with HFrEF at admission and discharge, along with reasons for not achieving the target dose from December 2023 to April 2024 (N = 215).

Drug Class (N)	Achieved TD	≥50–<100 % TD	<50 % TD	Eligible Patients	Reason Not Achieved TD	Frequency (%)
ACEI (N = 157)	100 (63.7 %)	0 (0 %)	57 (36.3 %)	168	Still in the up-titration	31 (18.5 %)
					Symptomatic hypotension	13 (7.7 %)
					No clear medical reason	5 (3.0 %)
					Renal disease	7 (4.2 %)
ARB (N = 5)	3 (60 %)	0 (0 %)	2 (40 %)	5	Still in the up-titration	1 (20 %)
					Symptomatic hypotension	1 (20 %)
					No clear medical reason	0
ARNI (N = 2)	1 (50 %)	0 (0 %)	1 (50 %)	2	Still in the up-titration	1 (50 %)
					Symptomatic hypotension	0
MRA (N = 140)	136 (97.1 %)	4 (2.9 %)	0 (0 %)	173	Still in the up-titration	2 (1.2 %)
					No clear medical reason	1 (0.6 %)
					Other reasons	1 (0.6 %)
BB (N = 139)	0 (0 %)	19 (13.7 %)	120 (86.3 %)	159	Still in the up-titration	29 (18.2 %)
					Bradycardia	16 (10.1 %)
					Symptomatic hypotension	28 (17.6 %)
					No clear medical reason	66 (41.5 %)
SGLT2i (N = 31)	31 (100 %)	0 (0 %)	0 (0 %)	187	–	–

**Footnotes:** TD = target dose. Patients who achieved the full recommended dose (≥100 % TD) were considered to have good guideline adherence. Those receiving ≥50 % but <100 % of the target dose were considered to have moderate adherence, while patients receiving <50 % of the target dose were classified as having poor adherence. Reasons for not achieving the target dose include clinical factors such as symptomatic hypotension, bradycardia, or renal disease, ongoing up-titration, or no clear medical reason.

### 3.7 Patients’ adherence to medication and lifestyles

The MARS-5 measures patient adherence to heart failure medications, with a mean score of 21.49. 74.9 % of patients had scores of 20 or above, while 11.2 % had scores below 20. Most patients reported rarely forgetting to take their medications, altering their dose, stopping medication, missing doses, or taking less medication than instructed. However, a smaller percentage engaged in non-adherent behaviors (Table 5).

### 3.8 Determinants of prescription adherence to GDMT

The bivariate multinomial logistic regression analysis showed that insurance coverage, length of hospital stays, salt restriction, HFEF, HF syndrome, presence of comorbidity, number of comorbidities, duration of HF, and admission in the last year (p < 0.25) were variables associated with either a poor or good level of adherence. However, the multivariate multinomial logistic regression analysis showed that insurance coverage, length of hospital stay, and salt restriction were significantly associated with a good level of adherence. On the other hand, HFEF and the number of comorbidities were significantly associated with poor levels of adherence.

The odds of a good level of adherence to GDMT compared to a moderate level of adherence to GDMT were about 4.81 times higher among HF patients who have insurance coverage, in contrast to those who pay medication out of pocket [AOR: 4.81; 95 % CI (2.81–21.33), p = 0.001]. The odds of a good level of adherence to GDMT compared to a moderate level of adherence to GDMT were decreased by 10 % among HF patients who stayed a longer duration of hospitalization, in contrast to those who stayed a shorter duration of hospitalization [AOR: 0.90; 95 % CI (0.74–1.18), p = 0.005], or

The odds of a moderate level of adherence to GDMT compared to a good level of adherence to GDMT were increased by 90 % among HF patients who stayed a longer duration of hospitalization, in contrast to those who stayed a short duration of hospitalization [AOR: 0.90; 95 % CI (0.74–1.18), p = 0.005]. The odds of a good level of adherence to GDMT compared with a moderate level of adherence to GDMT were about 6.62 times higher among HF patients who had not consumed salt, in contrast to those who had consumed salt [AOR: 6.62; 95 % CI (1.86–22.67), p = 0.003].

With a one-unit increase in the number of ejection fraction (HFEF), the odds of a poor level of adherence to GDMT were increased by 1.07 times over a moderate level of adherence to GDMT [AOR: 1.07; 95 % CI (1.02–1.12), p = 0.005]. With a one-unit increase in the number of comorbidities, the odds of a poor level of adherence to GDMT were increased by 2.36 times over a moderate level of adherence to GDMT [AOR: 2.36; 95 % CI (1.72–5.32), p = 0.037] (Table 6).

**Table 6:** MARS-5 descriptive statistics among heart failure patients at JMC, Southwest Ethiopia (Dec-Apr 2024, N = 215).

MARS-5 items	Frequency n (%)						Mean (SD)	Media (range)
Question (n = 185)	Always	Often	Sometimes	Rarely	Never	Total (100 %)		
"I forgot to take them."	2(0.9)	13(6.0)	4(1.9)	127(59.1)	39(18.1)	86.0	4.02(0.78)	4(4)
"I alter the dose."	0	7(3.3)	4(1.9)	77(35.8)	97(45.1)		4.43(0.72)	5(3)
"I stopped taking them for a while."	1(0.5)	9(4.2)	7(1.9)	70(32.6)	98(45.6)		4.38(0.82)	5(4)
"I decide to miss out on a dose."	2(0.9)	7(3.3)	10(4.7)	61(28.4)	105(48.8)		4.41(.84)	5(4)
"I take less than instructed."	0	6(2.8)	11(5.1)	75(34.9)	93(43.3)		4.38(0.74)	5(3)
MARS-5 adherence score (n = 185)	Frequency			Percentage (%)		86.0		
MARS score							21.49(3.32)	22(21)
Score (<20)	24			11.2				
Score ( ≥ 20)	161			74.9				

**Footnotes:** MARS-5 (Medication Adherence Report Scale-5) assesses patient adherence to prescribed medications. Each item is scored from 1 (“Always”) to 5 (“Never”), with higher scores indicating better adherence. The total MARS-5 score ranges from 5 to 25, where scores ≥20 reflect good adherence and scores <20 reflect poor adherence.

## 4 Qualitative findings components

### 4.1 Qualitative findings: physician perspectives on GDMT adherence

To explore the barriers and facilitators to guideline-directed medical therapy (GDMT) for HFrEF, semi-structured interviews were conducted with 12 physicians, including cardiologists, internists, and a clinical pharmacist, all with 2–15 years of clinical experience in managing HF at Jimma Medical Center. Responses were analyzed thematically, and the main themes are summarized in Table 7.

**Table 7:** Multivariate multinomial logistic regression analysis of determinants of prescription adherence in GDMTHeart Failure Patients at JMC, Southwest Ethiopia (Dec-Apr 2024, N = 215).

Variables	Category	Level of adherence					
		Good			Poor		
		AOR	95 % CI	p-value	AOR	95 %CI	p-value
Payer	Insurance	4.81	2.83–21.33	**0.001**	0.45	0.1–2.01	0.298
	Out pocket	1			1		
	Others	0.08	0.01–1.13	0.078	0.59	0.02–14.73	0.751
LOHS	Continues data	0.90	0.74–1.18	**0.005∗**	1.07	0.98–1.18	0.135
Salt restriction	Yes	6.62	1.86–22.67	**0.003∗**	1.62	0.40–6.55	0.499
	No	1			1		
HFEF	–	0.99	0.96–1.04	0.840	1.07	1.02–1.12	**0.005∗**
HF syndrome	De novo	1			1		
	Chronic ADHF	1.17	0.22–6.30	0.854	0.35	0.07–1.86	0.226
Comorbidity	Yes	0.19	0.02–1.90	0.158	2.2	0.05–20.08	0.994
	No	1			1		
HHD	Yes	3.82	0.75–19.49	0.106	0.02	0.09–4.76	0.699
	No	1	1		1		
No. comorbidity	Continues data	1.34	0.35–1.63	0.446	2.36	1.72–5.31	**0.037**
Duration of HF	Continues data	1.01	0.99–1.03	0.391	1.28	0.10–1.2.72	0.062
Since the last admission	Continues data	1.49	0.64–3.49	0.367	0.38	0.12–1.21	0.102

N/B: Moderate adherence to GDMT is the reference category in this model.

Keys: 1: reference variable category; COR: crude odds ratio; CI: confidence interval; AOR: adjusted odds ratio; HF: heart failure; HHD: hypertensive heart disease No.; Number of; LOHS, HFEF; heart failure ejection fraction, and ADHF; acute decompensated heart failure.

**Table 8:** Summary of themes, subthemes, and illustrative quotations on physician adherence to GDMT for HFrEF at Jimma Medical Center (Dec-Apr 2024, N = 12).

Theme	Subtheme/Barrier or Facilitator	Illustrative Quotation	Number of Participants Mentioning (n/N)
1. Familiarity with and Knowledge of the Guidelines	1.1 Awareness of the ESC Guidelines	“We now have a simplified, locally-tailored protocol based on ESC principles, which makes the guidelines practical rather than theoretical.” – Cardiologist 3	12/12
	1.2 Use of Local Adaptations	“Simplified local guidelines improve feasibility and relevance in routine practice.” – Internist 2	10/12
2. Barriers to Prescribing GDMT	2.1 Medication Unavailability	“We often face stock-outs of essential medications, especially ARNI and SGLT2 inhibitors.” – Internist 1	8/12
	2.2 Financial Barriers	“Many patients cannot afford the full treatment despite hospital support.” – Cardiologist 2	9/12
	2.3 Clinical Complexity/Contraindications	“In patients with severe renal impairment or low blood pressure, we must carefully modify the treatment plan.” – Cardiologist 1	7/12
3. Facilitators Supporting Guideline Adherence	3.1 Institutional/Administrative Support	“Support from hospital administration and reliable procurement enables adherence.” – Internist 3	10/12
	3.2 Multidisciplinary Collaboration	“Collaboration between physicians, pharmacists, and nurses improve patient outcomes.” – Cardiologist 4	9/12
	3.3 HF nurses' involvement	“Having a heart failure nurse to educate and follow-up with patients has been invaluable for long-term management.” – Clinical Pharmacist 1	8/12
4. Clinical Decision-Making and Justified Deviations	4.1 Patient-centered Decisions	“We rarely deviate from the guidelines now, and when we do, it's with a clear plan to get patients back on target therapy.” – Internist 2	7/12
	4.2 Temporary Adjustments with Optimization Plans	*“*Short-term modifications are implemented safely with planned re-initiation of full GDMT.” – Cardiologist 2	6/12
5. Patient-Specific Challenges	5.1 Economic Limitations	“Financial limitations remain a barrier for optimal therapy.” – Internist 1	9/12
	5.2 Comorbidities and Lifestyle	“Complex comorbid profiles and patient behaviors often require individualized care.” – Cardiologist 2	8/12
	5.3 Need for Continuous Patient Education	“Ongoing education about medications and lifestyle is essential for adherence.” – Clinical Pharmacist 1	10/12
6. System-Level Influences	6.1 Hospital Policies and Procurement	“Efficient hospital procurement policies directly influence prescribing practices.” – Internist 3	8/12
	6.2 Insurance and subsidy support	“Reliable insurance or subsidy schemes increase access to GDMT.” – Cardiologist 3	7/12
7. Recommendations for Improvement	7.1 Dedicated HF Clinics	“A specialized clinic would improve the continuity of care and allow structured follow-up.” – Cardiologist 4	9/12
	7.2 Continuous Evaluation of the Guidelines	“Ongoing local research helps evaluate and optimize guideline implementation.” – Internist 2	6/12

Notes:

• GDMT**:** Guideline-directed medical therapy.

• HFrEF: Heart Failure with Reduced Ejection Fraction.

• ESC: European Society of Cardiology.

**Summary:**The qualitative findings highlight the multilevel factors influencing physician adherence to GDMT in HFrEF. While knowledge, institutional support, and teamwork facilitate adherence**,** barriers such as medication shortages, patient financial constraints, and clinical complexity remain. These insights inform targeted interventions, including dedicated HF clinics, structured training, and policy support**,** to optimize guideline-based HF management in resource-limited settings.

#### 4.1.1 Key findings

Physicians identified multifactorial factors influencing adherence to GDMT, including prescriber knowledge, patient challenges, and system-level influences.

⁃ Familiarity with the Guidelines: Physicians were aware of the 2023 ESC heart failure guidelines and used locally adapted, simplified protocols to facilitate clinical implementation.
⁃ Barriers: Key barriers included medication unavailability, financial constraints for patients, and clinical complexity such as comorbidities and contraindications.
⁃ Facilitators: Institutional support, multidisciplinary teamwork, and dedicated heart failure nursing reinforced adherence to guideline-based care.
⁃ Clinical Decision-Making: Deviations from the guidelines were rare, patient-centered, and often short-term with a plan for optimization.
⁃ Patient-Specific Challenges: Economic limitations, comorbidities, lifestyle factors, and the need for ongoing education influenced treatment adherence.
⁃ System-Level Influences: Hospital policies, procurement efficiency, and insurance or subsidy schemes affected access to GDMT.
⁃ Recommendations: Physicians advocated for dedicated heart failure clinics and ongoing local evaluation of guideline implementation to improve patient outcomes (see Table 8).

## 5 Discussion

This mixed-methods study evaluated adherence to guideline-directed medical therapy (GDMT) for heart failure with reduced ejection fraction (HFrEF) at Jimma Medical Center, Ethiopia, integrating quantitative adherence measures with qualitative insights from healthcare providers. Our findings reveal that adherence to GDMT remains suboptimal, shaped by a complex interplay of patient, provider, and health system factors.

Quantitatively, only 16.3 % of patients were discharged on the full complement of recommended GDMT classes, indicating substantial gaps in prescriber adherence. Prescription rates for ACE inhibitors or angiotensin receptor blockers (73 %), mineralocorticoid receptor antagonists (67 %), beta-blockers (56 %), and sodium-glucose cotransporter-2 (SGLT2) inhibitors (14 %) reflect moderate initiation but suboptimal comprehensive treatment. Patient self-reported medication adherence was relatively high (74.9 %), yet adherence to lifestyle modifications, particularly salt restriction and physical activity, was poor. This discrepancy highlights the challenges in achieving the holistic guideline adherence necessary for optimal heart failure management [11,32,33]. Approximately 2 %–3 % of patients received ARBs or ARNIs, indicating low uptake due to barriers such as cost, availability, and clinical limitations such as hypotension and renal function issues. This pattern is consistent in resource-constrained settings, where challenges in affordability and supply chain affect the adoption of GDMT, despite evidence supporting ARNI regimens. The findings underscore the need for improved procurement, subsidy strategies, and clinician support to enhance access to ARNI therapy and align heart failure care with international standards.

Qualitative findings provide a critical context that explains and enriches these quantitative results through triangulation, enhancing the validity of our conclusions. Physicians consistently identified systemic barriers such as frequent stock-outs of essential medications, especially newer agents such as SGLT2 inhibitors, and financial constraints faced by patients. These factors directly contribute to incomplete prescriptions and hinder full guideline implementation [34,35]. Insurance coverage emerged as a strong facilitator, both quantitatively and qualitatively, with insured patients more likely to receive complete GDMT. Providers emphasized how expanded insurance schemes and subsidies improve access to medications and encourage guideline adherence, underscoring the essential role of financial protection in improving care delivery [[36], [37], [38]].

Longer hospital stays were associated with lower prescriber adherence, likely reflecting the increased clinical complexity and severity among patients requiring extended care. This was corroborated qualitatively, as physicians reported the need for individualized treatment plans and occasional deviations from guidelines due to comorbidities, contraindications, or acute clinical instability [39]. This underscores the balance that clinicians must maintain between evidence-based protocols and patient-centered decision-making, suggesting that adherence metrics should be interpreted with the clinical context in mind.

Patient adherence to lifestyle modifications, especially salt restriction, was positively associated with GDMT adherence. Physicians highlighted the importance of multidisciplinary care teams, including heart failure nurses, in educating patients and reinforcing adherence behaviors. These findings emphasize that effective heart failure management extends beyond pharmacotherapy to include comprehensive lifestyle support, an aspect often under-addressed in resource-limited settings [[40], [41], [42]].

Conversely, a higher burden of comorbidities was linked to poorer GDMT adherence, reflecting the challenges of managing polypharmacy, drug interactions, and competing clinical priorities. This aligns with qualitative reports of complex decision-making and highlights the urgent need for integrated, patient-centered care approaches to address multimorbidity in heart failure [43,44].

These results are consistent with those of prior research in Ethiopia and other low- and middle-income countries reporting low rates of full GDMT adherence among HFrEF patients. Prescription rates for ACE inhibitors/ARBs and MRAs in our study fall within the ranges reported in similar contexts (60–75 % for ACEI/ARBs; 50–70 % for MRAs), confirming persistent challenges in guideline implementation in resource-constrained settings [14,45,46]. However, the low SGLT2 inhibitor prescription (14 %) contrasts with higher rates reported in high-income countries (>30 %), likely reflecting disparities in drug availability, cost, and prescriber familiarity, as noted in other regional studies [47,48].

These relatively high patient self-reported medication adherence (∼75 %) differs somewhat from reports in similar populations, which often indicate lower adherence (<60 %). This may be due to differences in adherence assessment methods or the social desirability bias inherent in self-reporting. Nonetheless, poor lifestyle adherence aligns with the existing literature highlighting behavioral modification as a persistent challenge [11].

The positive association between insurance coverage and adherence is supported by previous studies demonstrating the beneficial impact of health insurance on medication access and chronic disease outcomes in comparable settings [49]. These qualitative data adds nuance by capturing prescriber perspectives on financial barriers, reinforcing the critical role of insurance.

The inverse relationship between comorbidities and adherence concurs with the literature indicating that multimorbidity complicates heart failure management, often necessitating guideline deviations [49]. The current mixed-methods approach uniquely illuminates clinicians’ balancing act between standardized care and individualized treatment, a complexity less explored in earlier quantitative-only studies.

The observed underutilization of ARNI and SGLT2 inhibitors in our cohort is concerning given their proven benefits in HFrEF. Contributing factors included high medication costs, limited local availability, and clinical contraindications. GDMT adherence scoring provided a quantitative measure of these gaps, demonstrating that even when patients were eligible, full target doses were often not achieved. These findings highlight the need for strategies to improve access, optimize titration, and enhance the implementation of guideline-directed therapies in routine clinical practice.

Overall, while these findings confirm many known patterns of GDMT adherence barriers in low-resource settings, they also highlight specific contextual factors at JMC, particularly drug availability and insurance coverage that shape adherence. These insights provide valuable evidence for locally tailored interventions.

The qualitative interviews conducted in this study provide important insights into the barriers and facilitators of heart failure management in a resource-limited setting. These findings underscore several priority areas for intervention. Strengthening medication procurement and supply chains is critical to ensure the consistent availability of essential GDMT drugs. Expanding health insurance coverage and aligning reimbursement policies with guideline recommendations can reduce financial barriers that limit both patient access and prescriber adherence. Enhancing healthcare provider training on GDMT and complex case management is essential. Establishing multidisciplinary heart failure clinics and investing in dedicated nursing sties study's qualitative interviews reveal key barriers and facilitators to managing heart failure in resource-limited settings. Barriers include high medication costs, limited access to recommended therapies, clinical contraindications, adverse effects, and patient challenges such as poor adherence and lack of understanding. Healthcare system issues, such as inadequate follow-up and staffing, further hinder management. Conversely, facilitators include effective patient education, strong support systems, structured follow-ups, and simplified treatment protocols, which positively impact medication adherence. The findings emphasize the interrelation between contextual barriers and adherence measures, providing insights for targeted interventions to enhance heart failure care in low-resource environments, which can improve patient education, adherence monitoring, and lifestyle modification [[48], [49], [50]].

### 5.1 Strengths and limitations

The strength of this study is its mixed-methods design, which allowed for the triangulation of qualitative provider perspectives with quantitative adherence data, yielding a more comprehensive and nuanced understanding of adherence issues. The findings’ robustness is further supported by the use of rigorous qualitative research and established adherence metrics. The cross-sectional design, which limits causal inference; the single-center design, which may limit generalizability; and the dependence on self-reported patient adherence, which is subject to bias, are some of the limitations. Furthermore, there were interpretive difficulties when combining several data sources; however, this was lessened by thorough triangulation.

## 6 Conclusions

Prescriber adherence to guideline-directed therapy for heart failure with reduced ejection fraction at Jimma Medical Center was inadequate, with limited patients receiving full recommended treatments. While good medication adherence was reported, lifestyle adherence, especially concerning salt restriction and physical activity, was poor. Factors such as insurance coverage, shorter hospital stays, and lifestyle adherence were associated with better prescribing practices. Conversely, higher ejection fraction and multiple comorbidities correlated with poorer adherence. Systemic barriers, including medication shortages, financial issues, and clinical complexities, were highlighted. To improve adherence to evidence-based care, enhancing procurement, expanding financial protection, increasing provider training, and establishing multidisciplinary heart failure services are necessary, which could significantly improve clinical outcomes in resource-limited settings.

## CRediT authorship contribution statement

**Getachew Yitayew Tarekegn:** Writing – review & editing, Writing – original draft, Project administration, Conceptualization. **Legese Chelkeba:** Writing – review & editing, Writing – original draft, Visualization, Validation, Conceptualization. **Mariam Dubale Deko:** Writing – review & editing, Writing – original draft, Visualization, Validation, Conceptualization. **Fisseha Nigussie Dagnew:** Writing – review & editing, Writing – original draft, Visualization, Formal analysis. **Samuel Berihun Dagnew:** Writing – review & editing, Writing – original draft, Methodology, Investigation, Conceptualization. **Tilaye Arega Moges:** Writing – review & editing, Writing – original draft, Validation, Data curation, Conceptualization. **Sisay Sitotaw Anberbr:** Writing – review & editing, Writing – original draft, Supervision, Conceptualization. **Taklo Simeneh Yazie:** Writing – review & editing, Writing – original draft, Data curation, Conceptualization. **Behailu Terefe Tesfaye:** Writing – review & editing, Writing – original draft, Visualization, Conceptualization.

## Ethics approval and consent to participate

The study was approved by the Institutional Review Board of Jimma University (Ref. No. JUIH/IRB/694/23). Written informed consent was obtained from the physicians participating in the qualitative interviews. Patient data were collected retrospectively, and confidentiality was maintained throughout the study. Participation was voluntary, and anonymity of the participants was ensured. The study adhered to the principles of the Declaration of Helsinki.

## Consent for publication

Not applicable. The manuscript does not contain any individual person's data in any form (including individual details, images, or videos).

## Availability of data and materials

The raw data supporting the findings of this study are available from the corresponding author upon reasonable request.

## Funding

The authors declare that they did not receive any funding for this research work.

## Declaration of competing interest

The authors declare that they have no competing interests. There were no financial or commercial relationships that could be understood as a potential conflict of interest.

## Abbreviations

ACEI: Angiotensin-converting enzyme inhibitor
ARB: Angiotensin receptor blocker
ARNI: Angiotensin receptor-neprilysin inhibitor
BB: Beta-Blocker
CRVDH: Chronic Rheumatic Valve Disease
DCMP: Dilated cardiomyopathy
ESC: European Society of Cardiology
GDMT: Guideline-directed medical therapy
HF: Heart Failure
HFrEF: Heart Failure with Reduced Ejection Fraction
HHD: Hypertensive Heart Disease
JMC: Jimma Medical Center
LOHS: Length of Hospital Stay
LVEF: Left Ventricular Ejection Fraction
MARS-5: Medication Adherence Report Scale–5 items
MRA: mineralocorticoid receptor antagonist
NYHA: New York Heart Association
SGLT2i: Sodium-Glucose Cotransporter-2 Inhibitors
