# Supplementary material for: Assessment of prescriber adherence to guideline-directed medical therapy for heart failure at Jimma Medical Center, Ethiopia
Source: Int J Cardiol Cardiovasc Risk Prev. 2025 Dec 5;28:200555. doi: 10.1016/j.ijcrp.2025.200555 (PMC12743505; doi:10.1016/j.ijcrp.2025.200555)
Supplement: Multimedia component 1 [file mmc1.docx]

## Supplementary file

## Guideline Therapy Use in HFrEF

This figure illustrates the distribution of prescription adherence based on the Guideline Adherence Index (GAI) and QUALIFIES criteria, and the frequency and percentage of major GDMT drug-class combinations prescribed before discharge and at discharge. The adherence component presents proportions categorized as good, moderate, or poor, while the combination-therapy component shows prescribing patterns across commonly used regimens, including ACEI/ARB/ARNI with beta-blockers, MRAs, and SGLT2 inhibitors ().

Figure 1: Distribution of GDMT Prescription Adherence Levels and Common Drug Class Combinations among Hospitalized HFrEF Patients (N = 215).

***Footnotes***

***Series 1:*** *Pre-discharge frequency****Series 2:*** *Discharge frequency****Series 3:*** *Pre-discharge percentage****Series 4:*** *Discharge percentage****Series 5:*** *QUALIFY good adherence****Series 6:*** *QUALIFY moderate adherence****Series 7:*** *QUALIFY poor adherence*

***ACEI:*** *Angiotensin-converting enzyme inhibitor****ARB:*** *Angiotensin II receptor blocker****ARNI:*** *Angiotensin receptor–neprilysin inhibitor****BB:*** *Beta-blocker****MRA:*** *Mineralocorticoid receptor antagonist****SGLT2i:*** *Sodium–glucose cotransporter-2 inhibitor****GAI:*** *Guideline Adherence Index****QUALIFY:*** *Quality Indicator for Prescription of Heart Failure Therapy*

## Prescription Adherence by Drug Class

This bar chart presents prescription adherence data from 215 participants in the GAI/QUALIFIES study, displaying adherence categorized by drug class and level. Blue bars indicate prescription frequency, while red bars show the percentage of total prescriptions per category. Adherence is divided into good, moderate, and poor groups. The chart highlights that most prescriptions fall into the moderate adherence category, especially for ACEI+MRA+BB combinations, with fewer achieving good adherence across drug classes (Figure 2).

Figure 2: Distribution of Combined GDMT Drug Classes Prescribed at Discharge (n=150)

*Footnotes:*

1. *GAI/QUALIFY: Refers to the specific study or dataset from which prescription adherence data were collected.*
2. *N=215: Total number of participants or prescriptions analyzed in this study.*
3. *Frequency: Number of prescriptions or instances recorded for each category.*
4. *Percent: Percentage of total prescriptions represented by each category.*
5. *Drug Classes:*
   - *ACEI: Angiotensin-Converting Enzyme Inhibitor*
   - *MRA: Mineralocorticoid Receptor Antagonist*
   - *BB: Beta-Blocker*
   - *SGLTI: Sodium-Glucose Cotransporter-2 Inhibitor*
   - *ARB: Angiotensin II Receptor Blocker*
   - *ARN: Angiotensin Receptor-Neprilysin Inhibitor*
6. *Combined drug classes indicate prescriptions involving multiple medications from the specified classes.*
7. *Adherence categories (good, moderate, poor) are based on predefined thresholds for medication adherence in the study protocol.*

# Full Questionnaire: Assessment of Prescriber and Patient Adherence to Guideline-Directed Medical Therapy (GDMT) in Heart Failure

## ****Section 1: Sociodemographic Information (Patient)****

1. **Age:** _______ years
2. **Sex:**
   - Male
   - Female
3. **Residence:**
   - Urban
   - Rural
4. **Marital Status:**
   - Single
   - Married
   - Divorced
   - Widowed
5. **Educational Level:**
   - No formal education
   - Primary education
   - Secondary education
   - Higher education
6. **Occupation:**
   - Farmer
   - Merchant
   - Civil servant
   - Labor
   - Retired
   - Student
   - Self-employed
   - Other: ___________
7. **Health Insurance Coverage:**
   - Yes
   - No
8. **Behavioral Characteristics:**
   - Do you currently smoke cigarettes?
     - Yes
     - No
   - Do you currently chew khat?
     - Yes
     - No
   - Do you currently drink alcohol?
     - Yes
     - No
9. **Physical Activity:**
   - Do you engage in regular physical activity (≥150 minutes/week)?
     - Yes
     - No
10. **Salt Intake:**
    - Do you regularly consume salt above recommended limits?
      - Yes
      - No

## ****Section 2: Clinical Characteristics (Patient/Medical Record)****

1. **Type of Heart Failure:**
   - De novo HF
   - Chronic acute decompensated HF (ADHF)
2. **Ejection Fraction (EF) %:** _______
3. **NYHA Functional Class:**
   - II
   - III
   - IV
4. **Comorbidities Present:**
   - Yes (Specify): _______________
   - No
5. **Length of Hospital Stay (days):** _______
6. **Discharge Unit:**
   - Cardiac ward
   - Medicine ward
   - Other: _____________

## ****Section 3: Prescriber Adherence to GDMT (Medical Record/Prescription Data)****

1. Was the patient prescribed the following medication classes at discharge? (Tick all prescribed)

| **Medication Class** | **Prescribed (Yes/No)** | **Dose Titration Status (≥100%, 50–<100%, <50%)** |
| --- | --- | --- |
| ACE Inhibitors (ACEI) | [ ] Yes [ ] No | ___________ |
| Angiotensin Receptor Blockers (ARB) | [ ] Yes [ ] No | ___________ |
| Angiotensin Receptor-Neprilysin Inhibitors (ARNI) | [ ] Yes [ ] No | ___________ |
| Beta-Blockers (BB) | [ ] Yes [ ] No | ___________ |
| Mineralocorticoid Receptor Antagonists (MRA) | [ ] Yes [ ] No | ___________ |
| Sodium-Glucose Cotransporter-2 Inhibitors (SGLT2i) | [ ] Yes [ ] No | ___________ |

1. If target dose was not achieved, specify reason(s):
   - Symptomatic hypotension
   - Bradycardia
   - Renal dysfunction
   - Ongoing up-titration
   - Other: ________________

## ****Section 4: Patient Medication Adherence (Self-Reported MARS-5 Scale)****

Please indicate how often you engaged in the following behaviors in the past month:

| **Item** | **Always (1)** | **Often (2)** | **Sometimes (3)** | **Rarely (4)** | **Never (5)** |
| --- | --- | --- | --- | --- | --- |
| 1. I forgot to take my medication | [ ] | [ ] | [ ] | [ ] | [ ] |
| 2. I altered the dose of my medication | [ ] | [ ] | [ ] | [ ] | [ ] |
| 3. I stopped taking my medication for a while | [ ] | [ ] | [ ] | [ ] | [ ] |
| 4. I decided to miss out on a dose | [ ] | [ ] | [ ] | [ ] | [ ] |
| 5. I take less medication than instructed | [ ] | [ ] | [ ] | [ ] | [ ] |

Total MARS-5 score: _______ (Sum of responses, range 5–25)
Scores ≥ 20 indicate good adherence.

## ****Section 5: Patient Lifestyle Adherence****

1. Do you restrict your salt intake as per medical advice?
   - Yes
   - No
2. Do you engage in regular physical activity as per medical advice?
   - Yes
   - No

## ****Section 6: Healthcare Provider Survey (Quantitative)****

1. Are you familiar with the 2023 ESC heart failure guidelines?
   - Yes
   - No
2. Do you use any local adaptations or protocols for guideline implementation?
   - Yes
   - No
3. What are the main barriers you face in prescribing GDMT? (Check all that apply)
   - Medication stock-outs
   - Financial constraints of patients
   - Clinical contraindications (e.g., hypotension, renal impairment)
   - Lack of guideline knowledge
   - Other: _______________
4. What facilitators help improve GDMT adherence? (Check all that apply)
   - Institutional support/administration
   - Multidisciplinary collaboration
   - Heart failure nurse involvement
   - Continuous medical education
   - Other: _______________
5. Do you deviate from GDMT guidelines in clinical practice?
   - Rarely, with clear patient-centered reasons
   - Frequently
   - Never
6. What recommendations do you have to improve GDMT adherence?

## ****Section 7: Qualitative Interview Guide for Healthcare Providers****

### Introduction

Thank you for participating. This interview will explore your experiences with prescribing GDMT for heart failure patients, including barriers and facilitators.

### Questions:

1. **Guideline Familiarity and Use**
   - How familiar are you with the 2023 ESC heart failure guidelines?
   - Do you use any local adaptations? How do they influence your prescribing?
2. **Barriers to Prescribing GDMT**
   - What are the main challenges you face when prescribing GDMT?
   - Can you describe issues related to medication availability or supply?
   - How do financial constraints impact your prescribing decisions?
   - Are clinical complexities (comorbidities, contraindications) a significant barrier?
   - Can you share instances where you deviated from guidelines? Why?
3. **Facilitators to GDMT Adherence**
   - What helps you adhere to GDMT guidelines?
   - How does institutional support or administration affect adherence?
   - What role does multidisciplinary collaboration play?
   - How do heart failure nurses contribute to adherence and patient care?
4. **Patient-related Challenges**
   - What patient behaviors or characteristics challenge optimal GDMT adherence?
   - How do you address medication adherence, lifestyle changes, or educational gaps?
5. **Healthcare System Influences**
   - How do hospital policies, procurement, or insurance coverage influence prescribing?
   - What systemic changes could improve adherence?
6. **Recommendations for Improvement**
   - What interventions would improve prescriber adherence to GDMT?
   - What is your view on dedicated heart failure clinics or specialized teams?
   - How can training and guideline updates be supported?

### Closing

- Is there anything else you’d like to share about heart failure management or guideline adherence?
- Do you have any questions about this study or interview?
